# Thymoma‐associated myasthenia gravis: Clinical features and predictive value of antiacetylcholine receptor antibodies in the risk of recurrence of thymoma

**DOI:** 10.1111/1759-7714.13724

**Published:** 2020-11-03

**Authors:** Anna De Rosa, Marco Fornili, Michelangelo Maestri Tassoni, Melania Guida, Laura Baglietto, Loredana Petrucci, Antonio Chella, Franca Melfi, Marco Lucchi, Roberta Ricciardi

**Affiliations:** ^1^ Department of Clinical and Experimental Medicine, Neurology Unit University of Pisa Pisa Italy; ^2^ Department of Clinical and Experimental Medicine University of Pisa Pisa Italy; ^3^ Department of Cardiology Thoracic and Vascular Medicine, Unit of Pneumology University Hospital of Pisa Pisa Italy; ^4^ Department of Cardiology Thoracic and Vascular Medicine, Minimally Invasive and Robotic Thoracic Surgery, Robotic Multispecialty Center for Surgery University of Pisa Pisa Italy; ^5^ Department of Cardiology Thoracic and Vascular Medicine University Hospital of Pisa Pisa Italy

**Keywords:** Antiacetylcholine receptor antibody, myasthenia gravis, thymoma, thymoma recurrence

## Abstract

**Background:**

Thymoma‐associated myasthenia gravis (TAMG) is one of the subtypes of myasthenia gravis with autoantibodies against the acetylcholine receptor (AChR‐Ab). We analyzed the clinical features of our cohort of TAMG patients and the changes in AChR‐Ab titer before and after thymectomy in order to identify factors predicting thymoma relapses.

**Methods:**

We retrospectively assessed: age of MG onset, MG clinical status according to MGFA (Myasthenia Gravis Foundation of America), epoch of thymectomy, post‐thymectomy status, oncological features and surgical approach. AChR‐Ab dosages were measured both before and after thymectomy. Linear regression models were applied to identify clinical determinants of AChR‐Ab titers and the Cox regression model was fitted to estimate the factors associated with the risk of thymoma recurrence.

**Results:**

The study sample included 239 MG patients, 27 of whom experienced one or more recurrences (median follow‐up time: 4.8 years). The AChR‐Ab titers decreased after first thymectomy (*P* < 0.001); the decrease was more pronounced in female patients (*P* = 0.05), in patients diagnosed with MG at an older age (*P* = 0.003), and in those who had lower MG stage before surgery (*P* = 0.02) or higher Masaoka‐Koga stage (*P* = 0.005). The risk of relapse was closely linked with the age of the patient, the Masaoka‐Koga stage and the surgical approach.

**Conclusions:**

Presurgery levels of AChR‐Ab or their change after surgery were not associated with thymoma recurrence. The reduction of AChR‐Ab titers after thymectomy confirms an immunological role of thymoma in the pathogenesis of MG.

**Key points:**

- Significant findings of the study: Young MG patients with an advanced Masaoka staging score of the primary tumor who underwent thymectomy with approaches different from sternotomy and VATS should be monitored for high risk of recurrence.
- What this study adds: No other study has ever investigated the changes in AChR‐Ab titers before and after thymectomy in a large cohort of TAMG patients. The reduction of AChR‐Ab titers after thymectomy suggests an immunological role of thymoma in the pathogenesis of MG.

## Graphical abstract

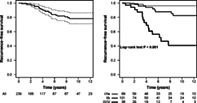

In our paper, we retrospectively analyzed the changes in antiacetylcholine receptor antibody (AChR‐Ab) titer before and after thymectomy and the clinical features of thymoma‐associated myasthenia gravis (TAMG) patients in order to identify clinical and serological predictors of thymoma recurrence. Our study emphasizes the importance of a multidisciplinary approach among Neurologists, Oncologists and Thoracic Surgeons, and identifies subgroups of MG patients with high risk of thymoma recurrence who therefore need a more accurate follow‐up.

## Introduction

Myasthenia gravis (MG) is the most frequent neurological disease associated with thymoma, constituting a real subtype of disease named TAMG (thymoma‐associated myasthenia gravis).^1^

Thymoma is a disease with malignant potential with a recurrence rate ranging from 5% up to 17% after complete resection.^2^ The average disease‐free time of recurrent thymoma is five years, but relapses 32 years after the initial thymectomy have also been reported.^3^ Thymoma recurrences are closely related to the World Health Organization (WHO) histological and Masaoka‐Koga classifications^2^, ^4^; relapsing thymomas, in fact, are more frequently type B2 and B3, according to the WHO classification, and belong to the more aggressive Masaoka‐Koga stages, such as stage IIb, III and IV. The most frequently reported relapses are those at pleural or otherwise intrathoracic sites, while distant metastases are rare.^5^

So far, to our knowledge, no other studies have been conducted to investigate the changes in AChR‐Ab titers before and after thymectomy in a large cohort of TAMG patients. Kim *et al*. analyzed AChR‐Ab titers in both thymomatous and nonthymomatous patients and they found a significant decrease in AChR‐Ab titer after thymectomy in nonthymomatous MG but not in patients with thymoma, suggesting that the pathogenic role of the thymus differs according to pathology.^6^

The aim of our study was to investigate the association of AChR‐Ab titers with the clinical characteristics of our cohort of TAMG patients and to evaluate their role in predicting thymoma recurrence.

## Methods

A retrospective analysis of a prospectively maintained database was conducted for all patients undergoing thymectomy for thymoma between January 1987 and December 2016 and regularly followed in the Clinic of Myasthenia Gravis and Thymus‐related disorders of the Department of Clinical and Experimental Medicine, Neurology Unit, and Division of Thoracic Surgery (Pisa). MG diagnosis was based on characteristic signs and symptoms of MG together with the anti‐AChR antibody positive test; patients with an anti‐AChR antibody negative test were excluded from the present study.

Surgical approaches were sternotomy, robotic‐assisted VATS, cervicotomy and thoracotomy. Patients who did not undergo a radical tumor excision according to surgical reports were excluded.

Patients were regularly followed up by the oncologist (A.C.) together with the neurologists, at intervals of three to four months for the first two years postoperatively, every six to eight months for the following three years and, then, annually or whenever it was required by the deterioration of the symptoms. A follow‐up CT scan was performed every year in the first five years after surgery and every two years thereafter to detect any recurrence of thymoma.

Age of MG diagnosis, MG clinical status, age of the patient at the time of thymectomy, time between age at MG onset and thymectomy, surgical approach and oncological features were retrospectively assessed from the medical records of all the patients.

MG clinical status was assessed before and one year following thymectomy according to MGFA classification.^7^ Thymomas were assessed according to the new WHO classification^8^ and the modified Masaoka‐Koga staging system.^9^

AChR‐Ab serum titers had been measured before and immediately after surgery by radio‐immune assay method (RIA).

Each patient gave an informed written consent for inclusion in the study that was conducted in accordance with the Declaration of Helsinki, and approved by the Ethics Committee of the Pisa University Hospital (Approval number 3470/2018).

The associations between the serological levels of AChR‐Ab and the clinical variables were assessed with linear regression models with the antibody concentrations before surgery (log transformed to achieve normality) or the differences after versus before surgery as dependent variables. The estimated marginal means (EMMs, that is the mean value of a variable predicted by the model for the mean value of each of the other variables included^10^) of the differences between the AChR‐Ab levels after versus before surgery and their standard errors were calculated for each level of the clinical characteristics from the model adjusted for the presurgery AChR‐Ab levels and from the model adjusted for all clinical variables.

The associations between the risk of worsening of the MG condition within one year after surgery and the clinical and serological variables were estimated by the logistic regression model, and the Wald test was used to assess the significance level of each predictor.

The median follow‐up time was obtained by the reverse Kaplan‐Meier method. The Kaplan‐Meier recurrence‐free survival probability was estimated at five and ten years after thymectomy and the Cox regression model was fitted to estimate the hazard ratio of recurrence for each clinical and serological predictor.

## Results

Table 1 shows the clinical and serological characteristics of the cases included in the study. Of the 239 patients, 121 (51%) were female. The mean age at MG onset was 49.8 years (SD = 13.8) while the mean age at thymectomy was 50.3 years (SD = 13.8). The mean time between age at MG onset and thymectomy was 0.45 years (SD = 3.1).

**Table 1:** Clinical and serological features of the patients

	Observed (%)
Sex	
Females	121 (51)
Males	118 (49)
Age at MG diagnosis (†)	50 (39–61)
Age at thymectomy (†)	50 (39–61)
MGFA status before thymectomy	
No symptoms/2A	74 (31)
2B	85 (36)
3/3A/4B/5	80 (33)
MGFA status after thymectomy	
No symptoms/2A	67 (28)
2B	133 (56)
3/3A/4B/5	39 (16)
Worsening of MG symptoms	
No	196 (82)
Yes	43 (18)
Masaoka‐Koga stage	
I/IIa	89 (39)
IIb	101 (45)
III/IV	36 (16)
WHO classification of thymoma	
AB	43 (19)
B1	78 (34)
B1/B2	41 (18)
B2/B3	69 (30)
Type of surgery	
Cervicotomy	3 (1)
Sternotomy	205 (87)
Robotic thymectomy	18 (8)
Thoracotomy	9 (4)
Radiotherapy or chemotherapy	
No	113 (47)
Yes	125 (53)
AChR‐Ab before thymectomy (†) (nmol/L)	9.2 (5.4–13.6)
AChR‐Ab after thymectomy (†) (nmol/L)	5.2 (2.5–9.7)

† Median (interquartile range).

Number of missing data: age at thymectomy *N* = 2; Masaoka‐Koga stage *N* = 13; WHO classification of thymoma *N* = 8; AChR‐Ab after thymectomy *N* = 99; type of surgery *N* = 4; radiotherapy or chemotherapy N = 1.

AChR‐Ab, acetylcholine receptor antibody; MG, myasthenia gravis; MGFA, Myasthenia Gravis Foundation of America; WHO, World Health Organization score.

Concerning surgery, 50 patients underwent thymectomy in other hospitals before our clinical evaluation. Due to the strong association between Masaoka‐Koga stage and the WHO classification of thymoma (*P* < 0.001), only the former was included in the multiple regression models.

### Variables associated with AChR‐Ab levels

The levels of AChR‐Ab before surgery were significantly higher in individuals with worse MGFA stage (*P* = 0.006); neither sex nor age at MG diagnosis nor Masaoka‐Koga stage were significantly associated with AChR‐Ab levels before surgery (not shown).

The AChR‐Ab titers decreased after first thymectomy (mean = −4.05; 95% CI: −5.12 to −2.97; *P* < 0.001). The reduction of the AChR‐Ab levels after surgery adjusted for before surgery levels are shown in Table 2 by individuals' characteristics: with respect to the AChR‐Ab levels before surgery, the reduction was significantly higher in females than in males (estimated marginal means = −4.66 vs. −3.25, *P* = 0.05), in cases diagnosed at older ages (−3.28 vs. −4.87 for those diagnosed before and after the age of 50 years, respectively, *P* = 0.003), in cases with lower MGFA stage (*P* = 0.02) and higher Masaoka‐Koga stage (*P* = 0.005) (Table 2).

**Table 2:** Relationship between patient characteristics and difference between the AChR‐Ab levels after and before thymectomy

	Model 1 (†)		Model 2 (‡)	
	EMMs (SE) (§)	*P*‐value	EMMs (SE) (§)	*P*‐value
Sex	_	*0.15*	_	0.05*
Females	−4.57 (0.52)	_	−4.66 (0.49)	_
Males	−3.49 (0.54)	_	−3.25 (0.51)	_
Age at MG diagnosis	_	*0.06*	_	0.003*
<50 years	−3.52 (0.46)	_	−3.28 (0.53)	_
≥50 years	−4.54 (0.45)	_	−4.87 (0.56)	_
MGFA status before thymectomy	_	*0.36*	_	0.02*
No symptoms/2A	−4.66 (0.71)	_	−5.19 (0.72)	_
2B	−4.32 (0.67)	_	−4.25 (0.74)	_
3/3A/4B/5	−3.41 (0.59)	_	−2.78 (0.66)	_
Worsening of MG symptoms	_	*0.91*	_	*0.37*
No	−4.03 (0.41)	_	−4.13 (0.38)	_
Yes	−4.14 (0.93)	_	−3.24 (0.89)	_
Masaoka‐Koga stage	_	0.04*	_	0.005*
I/IIa	−2.99 (0.60)	_	−2.53 (0.70)	_
IIb	−4.07 (0.56)	_	−3.96 (0.62)	_
III/IV	−5.46 (0.77)	_	−5.73 (0.79)	_
WHO classification of thymoma	_	*0.60*	_	_
AB	−4.15 (0.61)	_	NA	NA
B1	−3.57 (0.91)	_	NA	_
B1/B2	−3.53 (0.66)	_	NA	_
B2/B3	−4.98 (0.95)	_	NA	_

† Model 1, estimates from the model adjusted for AChR‐Ab before thymectomy. Analyses conducted on the individuals without missing values in the AChR‐Ab levels and in the corresponding predictor.

‡ Model 2, estimates from the model adjusted for AChR‐Ab before thymectomy and all the variables in the table except for the WHO classification of thymoma. Analysis conducted on 133 individuals without missing values in the AChR‐Ab levels and in any predictor.

§ EMMs, estimated marginal means of the difference of AChR‐Ab after minus before thymectomy;

AChR‐Ab, acetylcholine receptor antibody; MG, myasthenia gravis; MGFA, Myasthenia Gravis Foundation of America; NA: not applicable; SE, standard error; WHO, World Health Organization score.

*statistically significant.

### Variables associated with worsening of MG within one year after thymectomy

The only variable significantly associated with the risk of worsening of the MG symptoms was the MG stage (Table 3): the odds of getting worse were around 60% lower in those with a worse MG stage before surgery, although the association between worsening and MG stage was not statistically significant after the adjustment for all covariates. The results did not change after adjusting for type of surgery or adjuvant treatment (none or chemotherapy or radiotherapy) received after thymectomy (not shown).

**Table 3:** Association between worsening of myasthenia gravis (MG) symptoms within one year since the first thymectomy and clinical and serological characteristics

	Model 1 (†)		Model 2 (‡)	
	OR (95% CI)	*P*‐value (§)	OR (95% CI)	*P*‐value (§)
Sex	_	_	_	_
Males vs. females	0.97 (0.50–1.89)	*0.94*	0.65 (0.23–1.84)	*0.42*
Age at MG diagnosis (5‐unit increase)	0.94 (0.83–1.06)	*0.30*	0.97 (0.81–1.16)	*0.73*
MGFA status before thymectomy	_	0.007*	_	*0.11*
2B vs. no symptoms/2A	0.28 (0.12–0.66)	_	0.29 (0.07–1.11)	_
3/3A/4B/5 vs. no symptoms/2A	0.42 (0.19–0.92)	_	0.36 (0.11–1.16)	_
Masaoka‐Koga stage	_	*0.78*	_	*0.21*
IIb vs. I/IIa	1.07 (0.50–2.27)	_	2.05 (0.61–6.90)	_
III/IV vs. I/IIa	1.41 (0.54–3.69)	_	3.39 (0.86–13.34)	_
WHO classification of thymoma	_	*0.78*	_	_
B1 vs. AB	1.18 (0.47–2.99)	_	NA	NA
B1/B2 vs. AB	0.71 (0.30–1.71)	_	NA	_
B2/B3 vs. AB	0.96 (0.37–2.49)	_	NA	_
AChR‐Ab before thymectomy (nmol/L)	_	*0.24*	_	*0.72*
Tertile 2 vs. tertile 1	0.44 (0.13–1.44)	_	0.73 (0.21–2.55)	_
Tertile 3 vs. tertile 1	0.31 (0.07–1.36)	_	0.52 (0.10–2.62)	_
AChR‐Ab difference after minus before thymectomy (nmol/L)	_	*0.90*	_	*0.85*
Tertile 2 vs. tertile 1	0.73 (0.19–2.88)	_	1.01 (0.22–4.64)	_
Tertile 3 vs. tertile 1	0.85 (0.20–3.58)	_	1.39 (0.29–6.68)	_

† Model 1, unadjusted estimates except for AChR‐Ab before thymectomy and AChR‐Ab difference after versus before thymectomy, that are adjusted one for each other. Analyses conducted on the individuals without missing values in the corresponding predictor.

‡ Model 2, estimates adjusted for all the variables in the table except for the WHO classification of thymoma. Analyses conducted on 133 individuals without missing values in any predictor.

§ Wald test.

AChR‐Ab, acetylcholine receptor antibody; CI, confidence interval; MG, myasthenia gravis; MGFA, Myasthenia Gravis Foundation of America; NA: not applicable; OR, odds ratio; WHO, World Health Organization score.

*statistically significant.

### Variables associated with the risk of recurrence

**Figure 1:**
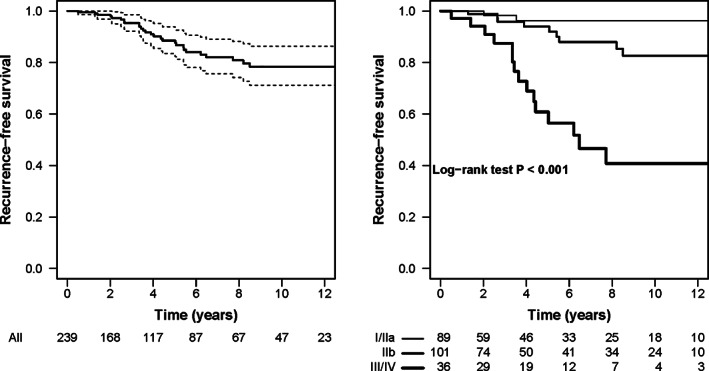
Left panel: Kaplan‐Meier estimate of the relapse‐free survival probability for all patients (solid line) with pointwise 95% confidence intervals (dashed lines). Right panel: Kaplan‐Meier estimate of the relapse‐free survival probability by Masaoka‐Koga stage (solid lines). In each panel the numbers of patients at risk are reported. Masaoka‐Koga stage: (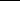) I/IIa, (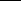) IIb, and (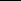) III/IV.

Out of the 239 patients, during a median follow‐up time of 4.8 years (interquartile range: 1.9 to 10.0 years), 27 relapsed. All first recurrences were pleural, except one paravertebral and one lung; moreover, four patients presented a recurrence both in pleural and diaphragmatic sites. Overall, the recurrence‐free survival probabilities at five and 10 years after thymectomy were 0.89, with 95% confidence interval (CI) 0.83–0.94, and 0.78 (95% CI: 0.71–0.86), respectively. The recurrence‐free survival probability was heterogeneous for Masaoka‐Koga stage (*P* < 0.001): the estimates were 0.96 (95% CI: 0.91–1.00), 0.94 (95% CI: 0.88–1.00) and 0.61 (95% CI: 0.45–0.82) at five years, and 0.96 (95% CI: 0.9–1.00), 0.83 (95% CI: 0.72–0.94) and 0.41 (95% CI: 0.25–0.67)) at 10 years for the Masaoka‐Koga stages I/IIa, IIb and III/IV, respectively (Fig 1).

The risk of recurrence was significantly associated with younger age (HR = 0.75 for a five‐year increase, 95% CI: 0.59–0.96, *P* = 0.02) and worse Masaoka‐Koga stage (HR = 10.18 for stages III/IV vs. I/IIa, 95% CI: 1.78–58.41, *P* = 0.007). Moreover, patients who underwent sternotomy or robotic thymectomy VATS experienced fewer recurrences than others (HR = 10.76 for cervicotomy/thoracotomy vs. sternotomy/robotic thymectomy, 95% CI: 2.42–47.82, *P* = 0.002, Table 4).

**Table 4:** Association between risk of recurrence after the first thymectomy and clinical and serological characteristics

	Model 1 (†)		Model 2 (‡)	
	HR (95% CI)	*P*‐value (§)	HR (95% CI)	*P*‐value (§)
Sex	_	_	_	_
Males vs. females	0.57 (0.26–1.25)	*0.16*	0.68 (0.25–1.86)	*0.46*
Age at MG diagnosis (for five years)	0.75 (0.64–0.89)	<0.001*	0.75 (0.59–0.96)	0.02*
MGFA status before thymectomy	_	*0.27*	_	_
2B vs. no symptoms/2A	0.69 (0.27–1.79)	_	_	_
3/3A/4B/5 vs. no symptoms/2A	1.49 (0.60–3.69)	_	_	_
MGFA status after thymectomy	_	<0.001*	_	*0.06*
2B vs. no symptoms/2A	0.33 (0.12–0.90)	_	0.20 (0.05–0.82)	_
3/3A/4B/5 vs. no symptoms/2A	2.85 (1.16–6.98)	_	0.60 (0.16–2.17)	_
Worsening of MG symptoms	_	_	_	_
Yes vs. No	1.31 (0.55–0.10)	*0.54*	0.42 (0.13–1.37)	*0.15*
Masaoka‐Koga stage	_	<0.001*	_	0.007*
IIb vs. I/IIa	3.66 (0.79–16.95)	_	2.07 (0.38–11.32)	_
III/IV vs. I/IIa	18.28 (4.18–80.06)	_	10.18 (1.78–58.41)	_
WHO classification of thymoma	_	0.04*	_	_
B1 vs. AB	2.41 (0.57–10.07)	_	NA	NA
B1/B2 vs. AB	5.15 (1.42–18.74)	_	NA	_
B2/B3 vs. AB	5.23 (1.41–19.33)	_	NA	_
AChR‐Ab before thymectomy (nmol/L)	_	*0.56*	_	*0.11*
Tertile 2 vs. tertile 1	0.60 (0.23–1.54)	_	0.31 (0.08–1.22)	_
Tertile 3 vs. tertile 1	0.80 (0.33–1.92)	_	0.20 (0.04–0.96)	_
AChR‐Ab difference after minus before thymectomy (nmol/L)	_	*0.99*	_	*0.45*
Tertile 2 vs. tertile 1	1.06 (0.37–3.01)	_	0.37 (0.08–1.77)	_
Tertile 3 vs. tertile 1	1.08 (0.44–2.66)	_	0.44 (0.09–2.16)	_
Type of surgery	_	_	_	_
Cervicotomy/thoracotomy vs. sternotomy/robotic thymectomy	5.88 (2.21–15.6)	<0.001*	10.76 (2.42–47.82)	0.002*

† Model 1, Cox regression model; unadjusted estimates. Analyses conducted on the individuals without missing values in the corresponding predictor.

‡ Model 2, Cox regression model; estimates adjusted for all the variables in the table except for MGFA status before surgery and WHO classification of thymoma. Analyses conducted on 133 individuals without missing values.

§ Wald test.

AChR‐Ab, acetylcholine receptor antibody; CI, confidence interval; HR, hazard ratio; MG, myasthenia gravis; MGFA, Myasthenia Gravis Foundation of America; NA: not applicable; WHO, World Health Organization score.

*statistically significant.

## Discussion

This study arises from the analysis of a large cohort of patients affected by thymoma‐associated myasthenia gravis with antiacetylcholine receptor antibodies.

Our data highlight a significant decrease of AChR‐Ab levels after intervention (*P* < 0.001). The relevant reduction of the antibody titer after surgery emphasizes the role of thymectomy as “disease modifying” treatment in MG with AChR‐Ab emphasizing that in patients with thymoma surgical radical treatment plays the same role as in patients with nonthymomatous MG.^11^

Presurgery levels of AChR‐Ab or their change after surgery were not associated with thymoma recurrence, although further studies with a larger sample size are warranted. So far, no clinical and serological biomarkers have been established to identify patients with a higher risk of tumor recurrence. Buckley *et al*. in a sample of 191 MG patients with thymoma and relapsing thymomas and without thymoma showed that interferon alpha and interleukin 12 increased substantially if thymoma recurred.^12^ In a previous study, we retrospectively collected data on 268 patients with thymomatous MG, and selected patients with symptoms of spontaneous muscle overactivity for autoantibody testing for neuronal cell‐surface proteins and cell‐based assays for contactin‐associated protein 2 (CASPR2), leucine‐rich glioma inactivated 1 (LGI1), glycine receptor and Netrin‐1 receptor antibodies. Neuromyotonia was diagnosed according to the presence of typical electromyography abnormalities and/or autoantibodies against LGI1/CASPR2. Accordingly, we found that thymoma recurrence was more frequent in those with than in those without neuromyotonia (*P* < 0.001).^13^

In our cohort of patients, we found that the risk of thymoma recurrence was higher in patients with early‐onset MG (*P* < 0.001 = 0.02). Recently, Tian *et al*. also found that older patients had a lower risk of recurrence (*P* = 0.009).^14^ We speculate that thymoma in the elderly arises from a thymic tissue that already underwent modifications towards apoptosis and tissue atrophy reducing its aggressiveness. Moreover, it is possible that in these patients the apoptotic effect of corticosteroids on the lymphocyte component of the tumors is more evident because of the longer MG history.^15^ This result indicates the necessity of a stricter surveillance in young patients with thymoma.

Antibody titers before thymectomy were significantly correlated with the severity of MG (*P* = 0.006) in accordance with literature data that show a direct correlation between antibody titer and MG severity.^16^, ^17^ Moreover, in our cohort, their decrease after surgery was greater in cases with lower MGFA stage before surgery (*P* = 0.02). We hypothesized that this subclass of thymoma patients could benefit from thymectomy more than others even if larger studies are needed to confirm this speculation.

Interestingly, we found that patients with a worse MG status (3/3A/4B/5 according to MGFA classification) before thymectomy experienced less MG exacerbations than others, within one year after thymectomy. This could be attributed to the beneficial effect on myasthenia gravis of higher doses of steroids administered in these patients in preparation for surgery to control myasthenic symptomatology. Furthermore, the literature data show that presurgical steroid treatment enhances the effectiveness of thymectomy^18^ and reduces the occurrence of relapses.^15^

Our data suggest that the recurrence‐free survival probability at 10 years is around 0.78 overall and 0.96, 0.83 and 0.41 in Masaoka‐Koga stages I/IIa, IIb and III/IV, respectively.

In our cohort of TAMG patients, 27 (11%) experienced one or more recurrences with a median follow‐up time after the first thymectomy of 4.8 years. These results are comparable with the study of the Japanese Association for Chest Surgery.^19^

In accordance with the literature data,^2^ in our study oncological characteristics of thymoma were closely associated with tumor recurrence. In particular, we identified advanced Masaoka staging score as a strong predictor of relapsing in the multiple Cox model (*P* = 0.007) while WHO histological type was evaluated only in the univariate model (*P* = 0.04) because of its tight relationship with Masaoka classification.^20^

In our cohort, sternotomy and robotic thymectomy VATS were associated with a lower risk of recurrence than thoracotomy and cervicotomy (*P* = 0.002). Recent reports indicated that for early‐stage thymoma there is not a significant difference between minimally invasive surgery and traditional open operation for experienced surgeons.^21^ However, it is of utmost importance to emphasize that the minimally invasive robotic thymectomy is a safe alternative to the median sternotomy approach in those forms of MG associated with thymic hyperplasia or noninvasive thymoma.^18^ Our study has some limitations and strengths. The data were analyzed retrospectively: in some patients the pre‐ and post‐thymectomy antibody titers were not measured in the same laboratory; this could explain, at least in part, the lack of statistical significance between the change of AChR‐Ab levels following thymectomy and thymoma recurrence. Furthermore, it is important to emphasize that 50 patients underwent thymectomy in other hospitals before our first clinical observation with surgical and therapeutic approaches different from ours. However, it should be emphasized that the number of the patients examined and followed homogeneously and the duration of follow‐up are strong elements of the study.

In conclusion, our study did not show association between AChR‐Ab dosages and the risk of thymoma recurrences in MG patients; conversely, it showed that young patients with an advanced Masaoka staging score of the primary tumor who underwent thymectomy with approaches different from sternotomy and VATS should be monitored for high risk of recurrence. The reduction of AChR‐Ab titers after thymectomy in MG patients with thymoma suggests an immunological role of thymoma in the pathogenesis of MG even if further studies are needed to confirm this hypothesis.

At present, since no clinical or laboratory factors to predict recurrence of thymoma are available, it should be good clinical practice to perform a careful multidisciplinary follow‐up (neurological, surgical, oncological) in patients with an aggressive histologic thymoma.

## Disclosure

The authors have no conflicts of interest to declare.
